# Developmental changes in abundance of the VSPβ protein following nuclear transformation of maize with the Soybean *vspβ *cDNA

**DOI:** 10.1186/1471-2229-5-3

**Published:** 2005-03-02

**Authors:** Magali F Grando, Rex L Smith, Cristina Moreira, Brian T Scully, Robert G Shatters

**Affiliations:** 1 Instituto de Ciências Biológicas/Faculdade de Agronomia e Medicina Veterinária, Universidade de Passo Fundo, Passo Fundo – RS, 99001-970, Brazil; 2 Agronomy Department, Institute of Food and Agricultural Science, University of Florida, Gainesville FL, USA; 3 United States Department of Agriculture, Agricultural Research Service, United States Horticultural Research Laboratory, 2001 South Rock Road, Fort Pierce, FL, USA; 4 Indian River Research and Education Center, Institute of Food and Agricultural Science, University of Florida, Fort Pierce, FL, USA

## Abstract

**Background:**

Developing monocots that accumulate more vegetative tissue protein is one strategy for improving nitrogen-sequestration and nutritive value of forage and silage crops. In soybeans (a dicotyledonous legume), the *vspA *and *B *genes encode subunits of a dimeric vegetative storage protein that plays an important role in nitrogen storage in vegetative tissues. Similar genes are found in monocots; however, they do not accumulate in leaves as storage proteins, and the ability of monocot leaves to support accumulation of an ectopically expressed soybean VSP is in question. To test this, transgenic maize (*Zea Mays *L. Hi-II hybrid) lines were created expressing soybean *vspB *from a maize ubiquitin *Ubi-1 *promoter.

**Results:**

From 81 bombardments, 101 plants were regenerated, and plants from five independent lines produced *vspB *transcripts and VSPβ polypeptides. In leaves from seven-week-old plants (prior to flowering), VSPβ accumulated to 0.5% of the soluble leaf protein in primary transgenic plants (R_0_), but to only 0.03% in R_1 _plants. During seed-filling (silage-stage) in R_1 _plants, the VSPβ protein was no longer detected in leaves and stems despite continued presence of the *vspB *RNA. The RNA transcripts for this peptide either became less efficiently translated, or the VSPβ protein became unstable during seed-fill.

**Conclusion:**

Developmental differences in the accumulation of soybean VSPβ when transgenically expressed in maize show that despite no changes in the *vspB *transcript level, VSPβ protein that is readily detected in leaves of preflowering plants, becomes undetectable as seeds begin to develop.

## Background

Although genetic variation for protein content has been found in forage plants, this variability is narrower than that observed for other traits such as digestibility [1]. Since the major protein components in monocot forage and silage crops are involved in metabolic activity, and hence are not "true" storage proteins, it has been argued that it is not feasible to make major changes in protein quality or protein composition by conventional breeding [1]. However, genetic engineering may allow improvement in protein quality and content through expression of a storage protein not found in grass vegetative tissue.

Genes encoding seed storage proteins of various plant species have been transgenically expressed to test for improvement of nutritional quality. Most experiments were conducted with tobacco and legume species including alfalfa, soybean, canola, clover and lupins. For nuclear-targeted genes, accumulation of these seed storage proteins in vegetative tissue of transgenic plants was either undetectable or very low. These included pea vicilin [2,3], soybean conglycinin [4], sunflower seed agglutinin [5,6], and phaseolin [7]. The instability of seed proteins in non-seed tissues of transgenic plants was frequently attributed to protein targeting to protease-rich vacuoles in the vegetative cells, and subsequent degradation [5,7,8].

The greatest accumulation of a seed storage protein from a nuclear-targeted gene was achieved using zeins, a maize seed storage protein that is targeted to "protein bodies" directly from the endoplasmic reticulum (ER), thus avoiding the secretory route to the cellular vacuole. Transgenic expression from the CaMV 35S promoter in tobacco resulted in the formation of these protein bodies containing the zein within vegetative tissues [8,9]. Alternatively, "short-circuiting" the protein-targeting route by addition of an ER retention signal to the storage-protein coding region also increased protein accumulation up to 100× [5,6,10,11].

In many legumes, accumulation of specific vegetative storage proteins (VSPs) in leaves and stems is the main source of increased nitrogen content [12-14]. Use of VSPs instead of seed storage proteins to increase vegetative protein content in monocots may provide an advantage since they have evolved to function in vegetative cell types. Legume vegetative cells that accumulate VSP proteins contain multiple vacuole types and storage proteins are targeted to specific vacuoles where they are not rapidly degraded [15], and do not interfere with cellular metabolic processes. It is uncertain if monocots can produce similar vacuoles or successfully target a VSP to them. The most studied VSPs are the soybean VSPα and VSPβ proteins, which are lysine-rich glycosylated vacuolar proteins that accumulate abundantly in leaves, stems and pods, but not in seeds [12-14]. Recently, soybean *vspA *and/or *vspB *genes fused to the 35S promoter were expressed in transgenic tobacco to study their accumulation in a heterologous dicot plant. Nuclear targeted genes produced VSP ranging between 2 and 6% of the soluble protein in leaves of the transgenic plants [16]; whereas, targeting to both the chloroplast and the vacuole within the same plant resulted in VSP comprising greater than 10% soluble protein [17,18]. Soybean VSP is therefore an excellent candidate for use in transgenic improvement of plant protein status, particularly grasses that contain limited levels of lysine [16]. However, it remains to be determined if VSPs can be expressed and accumulated in monocot plants since storage protein stability is dependent on post-translational events that may differ between monocots and eudicots. This manuscript presents the transgenic expression of soybean VSPβ in the leaves of transgenic maize and discusses this expression in relation to the developmental stage of the plant.

## Results and Discussion

### Development of primary (R_0_) transgenic maize expressing *vspB*

**Figure 1:**
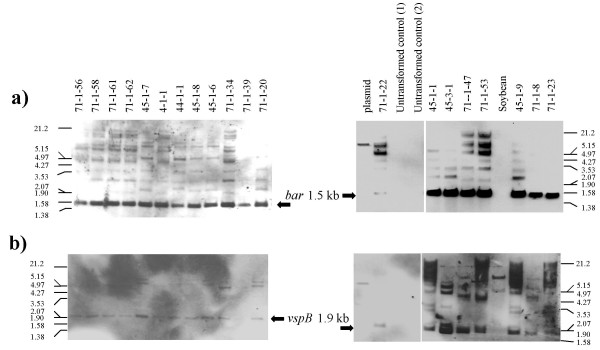
Southern blot analysis of primary (R_0_) maize transformed with pAHC25 and pRSVP-1. Twenty μgs of *Eco*RI restricted genomics DNA were electrophoresed through 0.8% agarose and blotted to Hybond membrane. The membranes were probed with either digoxigenin labeled *bar *(a) or *vspB *(b). Numbers and lines on sides of blots indicate location of molecular size markers (the number represents size in kilobases). The "a" and "b" sections are aligned so that the same genomic DNA samples are vertically aligned and represented by the same lane label. Plasmid lane is the plasmid containing the either the *bar *or the *vspβ *clones used as a positive control. Untransformed controls are lanes containing genomic DNA from untransformed Hi-II maize. Soybean indicates lanes containing restricted soybean genomic DNA. The two panels within each section represent separate blots hybridized with the same probe.

Out of 101 plants, twenty, belonging to five independent lines (71-1, 45-1, 45-3, 44-1, and 4-1) were shown by Southern blot analysis to contain a 1.5 kb hybridizing band corresponding to the intact *bar *gene (Fig. 1). All 20 plants also contained the expected 1.9 kb band that hybridized to the *vspB *gene. The same probe detected two bands in *Eco*RI restricted Soybean genomic DNA, ~5.7 and 8.6 Kb, corresponding to the highly homologous genes *vspA *and *vspB *[13].

**Figure 2:**
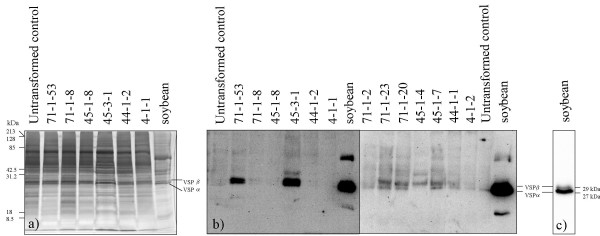
Western blot detection of VSPβ in primary (R_0_) transgenic maize. Thirty micrograms of protein from each sample were separated by SDS-PAGE. (a) Silver-stained 12% SDS-PAGE polypeptide profile for six of the 14 analyzed R_0 _plants. (b) Immunodetection of VSP protein in SDS-PAGE separated transgenic R_0 _maize extracts transferred to Hybond-P membranes and immunodetected using VSPβ antiserum and the Reinascence kit chemiluminescent detection method (NEN Life Sciences Products, Inc). (c) Underexposed western blot showing two distinct bands corresponding to VSPα and VSPβ polypeptides in soybean leaves.

Western blot analysis was used to detect the VSPβ polypeptide in leaf extracts from fourteen primary (R_0_) transgenic maize plants at vegetative stage (7 weeks old). A distinct VSPβ band was not visible in silver-stained SDS-PAGE separated maize extract proteins due to complexity of the total protein pattern and the relatively low level of VSPβ expression (Fig. 2a). However, some of these plants expressed VSPβ at a level high enough to be detected by Western blot analysis (Fig. 2b). Two plants (71-1-53 and 45-3-1) had the highest level of VSPβ, while four plants (71-1-23, 71-1-20, 45-1-4, and 45-1-7) accumulated a lower level of VSPβ. Although a faint lower MW peptide band was visible in negative control maize extracts, VSP was clearly only present in transgenic lines. Faint detection of bands in untransformed maize (none of identical size to soybean VSPβ) is consistent with previous reports of cross-hybridizing of soybean VSP antibodies with proteins from monocots [14]. In soybean extracts used as a positive control, the antibody for VSPβ recognized both VSPα and VSPβ polypeptides (Fig. 2b,c)

Computer analysis of digital images of the Western blots was used to detect differences in relative band intensity of the immunologically detected VSPβ peptide. Because the native soybean VSPs (VSPα plus VSPβ) were easily visible on total protein stained gels (Fig. 2a), relative quantification of total stained proteins in the soybean samples indicated that the VSPs represented about 10% of the total soluble protein in young soybean leaves. This is close to the previously reported value of 15 % [12]. The intensity of the transgenic maize 45-3-1 VSPβ band on Western blots was 44% of the soybean VSP's band (digital image pixel quantification of Fig. 2b). Accounting for the differences in total protein applied to the gel (less soybean total protein was loaded), the VSPβ protein was estimated to have accumulated to 0.5 % of the total soluble protein. This is similar to the highest level of seed storage protein accumulation observed with the ectopic expression of zein [8,9], but remains less than the 1% minimal expression level predicted by Wandelt et al. [11] to be needed to directly alter the nutritional quality of the leaves. Although the 0.5% of total soluble protein was too low to alter nutritional value, detection of VSPβ in 45-3-1 allowed monitoring of VSPβ level in leaves and stems during plant development.

### Presence of *vspB *in R_1 _plants

**Figure 3:**
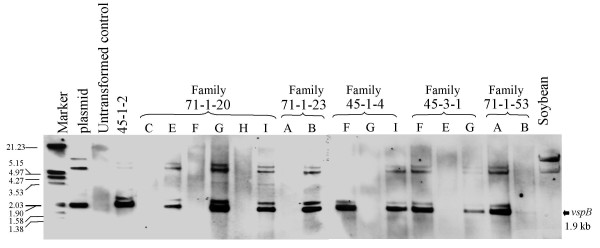
Southern blot detection of *vspB *in R_1 _transgenic maize. Twenty micrograms of *Eco*RI restricted genomics DNA was electrophoresed through 0.8% agarose and blotted to Hybond membrane. The membrane was probed with digoxigenin labeled *vspB*. The alphabetical labels of each R_1 _family represent individual R_1 _plants. Control sample represent genomic DNA from untransformed maize Hi-II. Two positive control samples are also included: plasmid, pRSVP-1 restriction digested to liberate the *vspB *cDNA clone (only partial digestion occurred as evidenced by the two bands at higher molecular weight than the *vspB *fragment), and genomic DNA from the 45-1-2 R_0 _plant, previously shown to contain the *vspB *sequence. The *Eco RI *restricted soybean genomic DNA is also included as a positive control for hybridization of the soybean *vspB *probe.

R_1 _plants were produced by back-crossing the R_0 _plants with Hi II control non-transformed pollen. Back-crossing was performed because R_0 _plants directly regenerated from tissue culture did not have synchronized production of pollen and receptive female flowers. The R_1 _families segregating for *bar *expression were analyzed for the presence of the *vspB *gene by Southern blot analysis. From 57 R_1 _plants analyzed, Southern blot analysis showed that 35 (61%) contained the *vspB *gene integrated into the genome. Figure 3 shows examples for several R_1 _from five R_0 _parental lines. The ~1.9 kb *vspB *band can be seen in nine of the 16 R_1 _lines. There are also fainter bands one slightly larger than the 1.9 kb band and one or two migrating between 4 and 5 kb. These are often observed as incomplete restriction of all *Eco RI *sites internal to plasmid DNA that is integrated into the maize genome. Similar bands are observed even with the plasmid control.

**Figure 4:**
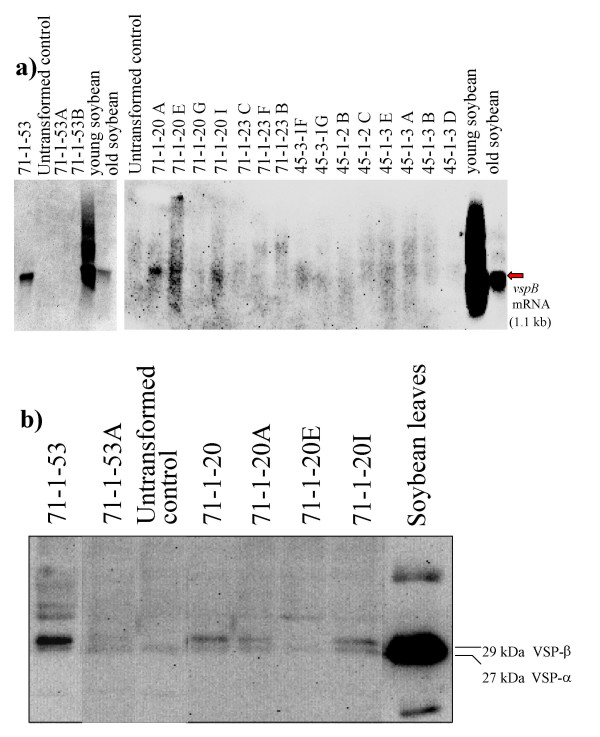
(a) Northern blot detection of *vspB *transcripts in R_1 _transgenic maize. Thirty micrograms of total RNA were separated on 1.2% agarose formaldehyde gels and blotted to Hybond N^+ ^membranes. The *vspB *transcripts were detected by hybridized with a digoxigenin labeled *vspB *probe. (b) Western blot detection of VSP in R_0 _and their progeny (R_1_) transgenic maize. Thirty micrograms of protein in extracts from leaves of 7 weeks old plants were separated on 12% SDS-PAGE, blotted onto Hybond-P membrane, and VSP was immunodetected using VSPβ antiserum and the Renascence kit chemiluminescent detection method (NEN Life Sciences Products, Inc).

Total RNA was isolated from leaves of young R_1 _plants at the vegetative stage (7 weeks after planting). Eighteen transgenic R_1 _plants, including the ones originating from R_0 _plants representing different levels of VSPβ accumulation (high VSPβ accumulators: 45-3-1, 71-1-53; mid-level VSPβ accumulators: and 71-1-23, 71-1-20, 45-1-4, 45-1-7 and low VSPβ accumulators: 44-1-1- 4-1-2), were analyzed for *vspB *transcripts. Because of high sequence similarity (85%) between *vspA *and *vspB *cDNAs, the same probe hybridized in soybean with both mRNAs as demonstrated by Staswick [13]. The transgenic plants 71-1-53A, 71-1-20A, 71-1-20E, and 71-1-20I produced a hybridizing band of approximately the expected size of 1.1 kb indicating transgene expression (Fig. 4a); however, most did not. Use of the *Ubi*-1 promoter for both the *vspB *and *bar *gene probably led to a high number of transgenics with the *vspB *silenced, and a higher level of VSPβ accumulation will likely result in future work using a combination of different promoters.

Immunodetection of VSPβ protein in transgenic young plant leaves showed variation in accumulation in comparisons between the parental (R_0_) and their R_1 _progeny, with the greatest variation observed with the highest VSPβ expressing R_0 _plants (Fig 4b). The parental line 71-1-53 had a relatively high level of expression of VSPβ, but the only transgenic offspring from this line, 71-1-53A, had almost undetectable VSPβ; in contrast, a low level accumulator, 71-1-20, produced R_1 _lines expressing different levels of VSPβ, although none of them expressed at higher levels than the parent. Quantification of the relative level of VSPβ expressed in the R_1 _leaves showed that the highest level measured was only 0.03% of total soluble protein.

**Figure 5:**
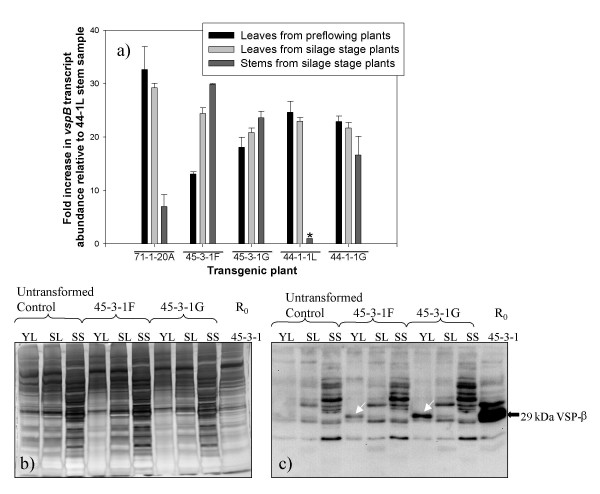
a) Real-time RT-PCR quantification of *vspB *transcripts in R_1 _transgenic maize tissue. Two hundred micrograms of total RNA from the indicated tissue was used as the template source for real-time RT-PCR detection of *vspB *transcripts. Reactions were performed in 15 uL volume using the Qiagen Quantitect SYBR Green kit. Expression values are calculated by normalizing all threshold cycles (C_t_) for *vspB *to the 18S rRNA C_t _and converting this value to fold-increase over the value for the lowest expressing tissue, *44-1-1-stem, which was arbitrarily set at 1.0. b,c) Developmental changes in VSPβ level in transgenic maize vegetative tissues. Soybean VSPβ was immunodetected in Western blots of 30 μgs of total protein from crude extracts separated by 12% SDS-PAGE and blotted to Hybond-P membranes. Crude Extracts were prepared from: YL, leaves from preflowering plants; SL, leaves from silage stage plants; and SS, stems from silage stage plants. (b) Coomassie blue stained SDS-PAGE separated proteins from crude extracts; (c) VSP was immunodetected using VSPβ antiserum and the Renascence kit chemiluminescent detection method (NEN Life Sciences Products, Inc). The arrows indicate the VSP-β protein band.

Despite the overall low level of VSPβ expression, for the purposes of this work, VSPβ accumulation in several of the plants was high enough to study the relationship of plant developmental stage and *vspB*/VSPβ accumulation. Both the transcript abundance and VSPβ protein accumulation were determined in the R_1 _lines using real-time quantitative RT-PCR and Western blot immunodetection, respectively. Real-time RT-PCR is more sensitive that Northern blot analysis and was able to detect transcripts that were not seen with standard total RNA blotting methods. The *vspB *transcript was quantified in leaves from immature plants (prior to tassel formation) and silage stage plants (plants with developing seeds at the 18 DAP-days after pollination stage), as well as, stems from the silage stage plants, (Figure 5). The *vspB *transcript was detected in all five transgenics (four of which had RNA not detectable using standard Northern blot methods). The relative level of RNA among the different plant samples was not consistent across the different lines with some having more transcripts in the young leaves while others had more in the older leaves and stems.

When *vspB *RNA abundance was compared to VSPβ protein accumulation there was no correlation between the two (Fig. 5a, b, c). Antiserum to VSPβ detected VSPβ only in leaves from young maize plants that had not yet flowered. No VSPβ was detected in leaves and stems of silage stage corn that had developing seed. The soybean VSPβ peptide was the primary band reacting with the antiVSPβ antiserum in young leaves of transgenic maize, however, in silage stage stems and to a lesser extent the silage stage leaves, there were multiple bands detected at a different size than the VSPβ. These were also detected in the non-transgenic control plant samples and therefore, were not due to different modifications of the soybean VSPβ. Although the VSPβ protein dropped below detectable levels in the silage stage 71-1-20A, 45-3-1F and 45-3-1-G plants, the *vspβ *transcript was still detectable. In fact, in the 71-1-20A plants the transcript level was highest in the silage stage leaves that had no detectable VSPβ. Therefore, post-transcriptional events (i.e., changes in either the translational efficiency of the *vspB *transcript or the protein stability of VSPβ) were altered in the silage stage leaves and stems as compared to the leaves of immature plants.

## Conclusion

The *vspB *gene was successfully introduced into R_0 _regenerated maize and transferred to the R_1 _progeny, of which *vspB *transcript and VSPβ protein were detected and studied. This is the first report on introduction and expression of a legume vegetative storage protein in a monocot plant. The inability to detect VSPβ from the maize vegetative tissue at the time of seed development, even when the *vspB *transcript was still expressed, must have arisen from either reduced translational efficiency of the *vspB *transcript or a decrease in the stability of the VSPβ protein. The reduction of seed storage protein level in leaves of transgenic eudicots was also observed with expression of vicillin in alfalfa [11] and tobacco [2]; however, it was not observed with the expression of VSPα in tobacco [16] or ovalbumin in alfalfa [30]. These data suggest that factors controlling developmental change in vegetative tissue protein accumulation are a combination of host plant traits and innate characteristics of the ectopically expressed protein. It is interesting to speculate that if, in maize (a monocot), soybean VSPβ was degraded in a manner that provided amino acids that were translocated to the seed to support seed development, then development of high level VSP expressing monocots may be a way to improve nitrogen content of the seed/grain produced by the plant.

## Methods

### Plant material and tissue culture methods

Plants of hybrid "Hi-II" maize were established in a greenhouse and immature tassels were used for embryogenic type II callus production, as described by Armstrong [19].

### Transgenic plant development

Microprojectile bombardment of callus was performed using the procedure of Somers et al. [20]. Calli were cobombarded with equal amounts of pRSVP-1 (Shatters Jr., unpublished) and pAHC25 [21]. Plasmid pRSVP-1 was constructed by restricting the soybean *vspB *cDNA clone (998 bp) from pKSH3 [22] with *Eco*RI, blunt ends of this fragment were produced using S1 nuclease and the fragment was cloned into similarly blunt ended *Bam*HI restricted pAHC17 [21]. As a result, the *vspB *coding region was inserted downstream of the *Ubi-1 *promoter and a 5' untranslated region (exon) and intron; and upstream of the *nos *terminator sequence. The plasmid pAHC25 carried the *bar *gene and the *uidA *reporter gene, both under the control of the *Ubi-1 *promoter. Bombardments were performed with a Biolistic^® ^PDS-1000/He Particle Delivery System (Bio-Rad Laboratories, Hercules, CA) and an osmotic treatment was applied to reduce the cell damage caused by the gene transfer method [23]. Putative transgenic maize were regenerated from glufosinate resistant callus as described by Armstrong [19], and grown in five-gallon pots containing sterile sand and Metromix-350 (1:1). Plants were fertilized weekly with Peter's 20-20-20 with micronutrients (Division of United Industry Corp., St. Louis, MO).

### Southern blot analysis

One gram of frozen young leaf tissue was ground in liquid nitrogen and genomic DNA was extracted using the Dellaporta procedure [24]. Twenty micrograms of genomic DNA were digested with *Eco*RI, which released a 1.9 Kb fragment containing the *vspB *gene, the *nos *terminator and part of the *Ubi-1 *promoter. DNA was separated on a 0.8% agarose gel, blotted onto Hybond N^+ ^membrane (Amersham Pharmacia Biotech, Inc. Piscataway, NJ) by capillary blotting [25], and UV cross-linked. The non-radioactive digoxigenin system (Roche Molecular Biochemicals, Indianapolis, IN) was used for labeling and detection of the transgene. Blotted DNA was probed with either a 611 or a 843 bp of *vspB *gene segment amplified from pRSVP-1 and gel purified. The forward and reverse primers 5'-GTTCTTCGGAG GTAAAAT-3' and 5'-TTCGCCTCTGTGGT-3' were used, respectively, to amplify a 611 bp segment, and the primer pair 5'-GCAGGCTACCAAAGGT-3' and 5'-TAGGTGACTTACCCACAT-3' was used to amplified the product of 843 bp.

For identification of *bar *transgenic plants, the DNA was digested with *Eco*RI, which released a fragment of ~1.5 Kb that contained part of the *Ubi-1 *promoter, the *bar *gene, and the *nos *terminator, and was identified with a 419 bp digoxigenin labeled probe produced by PCR amplification of pAHC25 using the forward and reverse primers: 5'-GGCGGTCTGCACCATCGT-3' and 5'-GCCAAGTTCCCGTGCTTGA-3', respectively.

### Northern blot analysis

Total RNA was isolated from 2 g of tissue using acid guanidine isothiocyanate-phenol-chlorophorm extraction [26], resuspended in T_10_E_1 _and treated with 2 μl RNasin^® ^(4U/μl) RNAse inhibitor (Promega, Madison, WI) and stored at -70°C until use. Thirty micrograms of total RNA were separated on 1.2% agarose formaldehyde gels and transferred to Hybond N^+ ^membrane by capillary blotting overnight or by pressure blotting for one hour using a PosiBlot^® ^30-30 pressure blotter (Stratagene, La Jolla, CA) according to the manufacture's instructions. Membranes were UV-crosslinked, and probed with the same probes as described for Southern blot analysis. Chemilluminescence was captured using a Kodak Digital Science Image Station 440 CF (Eastman Kodak Company, Rochester, NY).

### Real-Time RT-PCR

Total RNA extractions for real-time RT-PCR were performed using 500 mg of tissue ground to fine powder using mortar and pestle in the presence of liquid nitrogen, then processed with the RNeasy midiprep Kit (Qiagen, Germany), following the manufacturer's protocol. Trace DNA contamination was removed from total RNA by a combination of acid phenol: chloroform 5:1 pH= 4.7 extraction and Dnase I treatment (Ambion, Texas). Real-time RT-PCR was performed on a Rotor-Gene RG-3000 (Corbett Research, Australia) using the Quantitect SYBR Green real-time RT-PCR kit (Qiagen, Germany), and the manufacturers protocols with 300 ng of Dnase I treated total RNA. Primers were designed to amplify a 108 bp fragment of the soybean *vspB *using the following primers: 5'-TGGTTCAACGCACTCTTC-3' and 5'-GGCTATGGTGAGCGTTCTTC-3'. Reverse transcription was performed for 30 min at 50°C followed by a 15 min denaturing at 95°C, and 40 cycles of 40 s at 95°C, 40 s at 58°C and 40 s at 72°C. Quantification was based on relative abundance to maize 18S RNA by amplifying a 174 bp fragment with primers: 5'-CCTGCGGCTTAATTGACTC-3' and 5'-GTTAGCAGGCTGAGGTCTCG-3', and using the comparative quantification function of the Rotor-Gene RG-3000 software. All real-time RT-PCR experiments were conducted in triplicate and on triplicate RNA preparations for each sample. Melting curve analysis and agarose gel electrophoresis were performed to verify single product formation.

### Western blot analysis

Protein was extracted from 100 mg of leaves and stems with 0.5 ml of phosphate-buffered saline (137 mM NaCl, 2.7 mM KCl, 10 mM Na_2_HPO_4_, 2 mM KH_2_PO_4_) supplemented with 1 tablet/10 ml buffer of the Complete-Mini protease inhibitor cocktail (Roche Molecular Biochemicals, Indianapolis, IN) by homogenizing in the presence of zirconia/silica 0.1 mm dia. beads. Centrifuged extract supernatants were removed and used for protein concentration determination [27]. Because of the low protein yields in stem extracts, they were precipitated with 10% trichloroacetic acid (TCA), washed with ice-cold acetone and resuspended prior to SDS-PAGE analysis.

Thirty micrograms of protein were separated on 12% SDS-polyacrylamide gels. Proteins in the gel were either stained with silver nitrate [28] or transferred to Hybond-P (PVDF) membranes (Amersham Pharmacia Biotech, Inc. Piscataway, NJ) using Trans-Blot SD Semi-Dry Transfer Cell blotter and recommended protocols (Bio-Rad Laboratories). Soybean VSP was immunologically detected on the PDF membranes with anti VSPβ serum (provided by P. Staswick, University of Nebraska, produced as previously described [29] used at a 1:5,000 dilution. Detection was performed using a luminol substrate and the NEN Life Science Products, Inc. (Boston, MA) Reinascence kit. Chemiluminescent signal was captured by a Kodak Digital Science Image Station 440 Cf, and analyzed with Kodak 1D Scientific Image Software. Quantification of band intensity was always compared relative to samples from the same gel.

## List of abbreviations

RT-PCR, reverse transcriptase-polymerase chain reaction; VSP, vegetative storage protein

## Authors' contributions

MFG participated in experimental design, carried out the transgenic plant development, plant crosses, and molecular blotting/detection methods, and participated in manuscript draft preparation. RLS participated in experimental design, and provided guidance and training in development of transgenic maize. CM performed RT-PCR experiments. BTS participated in experimental design and provided expertise and training in plant crosses. RGS conceived of the study, participated in experimental design, coordinated the experimental plan, and wrote the draft manuscript. All authors read and approved the final manuscript.
